# Schizophrenia genomics and proteomics: are we any closer to biomarker discovery?

**DOI:** 10.1186/1744-9081-5-2

**Published:** 2009-01-07

**Authors:** Shaheen E Lakhan, Alon Kramer

**Affiliations:** 1 Global Neuroscience Initiative Foundation, Los Angeles, CA, USA

## Abstract

The field of proteomics has made leaps and bounds in the last 10 years particularly in the fields of oncology and cardiovascular medicine. In comparison, neuroproteomics is still playing catch up mainly due to the relative complexity of neurological disorders. Schizophrenia is one such disorder, believed to be the results of multiple factors both genetic and environmental. Affecting over 2 million people in the US alone, it has become a major clinical and public health concern worldwide. This paper gives an update of schizophrenia biomarker research as reviewed by Lakhan in 2006 and gives us a rundown of the progress made during the last two years. Several studies demonstrate the potential of cerebrospinal fluid as a source of neuro-specific biomarkers. Genetic association studies are making headway in identifying candidate genes for schizophrenia. In addition, metabonomics, bioinformatics, and neuroimaging techniques are aiming to complete the picture by filling in knowledge gaps. International cooperation in the form of genomics and protein databases and brain banks is facilitating research efforts. While none of the recent developments described here in qualifies as biomarker discovery, many are likely to be stepping stones towards that goal.

## Background

Schizophrenia is a complex disturbance of mind and brain characterized by psychotic symptoms such as delusions and hallucinations. In a review of several epidemiological estimates using varied criteria, McGrath et al. [1] report the worldwide incidence of schizophrenia to range from 7 to 42 per 100,000 population. In the US alone, over 2 million Americans have been diagnosed with schizophrenia [2]. The US Centers for Disease Prevention and Control (CDC) estimated the national expenditure for overall mental health in 2003 at over $100 billion.

The history of schizophrenia is obscure possibly due to the heterogeneous nature of the disorder and consequent difficulty in formal classification. Earliest case reports date back to the 1800s [3]. Despite an abundance of research, the pathogenesis and etiology of this often highly disabling disorder remains unclear. Not surprisingly, the diagnosis and treatment of schizophrenia remains problematic; mainly for the following reasons:

• Diagnosis is solely based on behavioral markers, either as self-reported symptoms by patients or observations by clinicians.

• No laboratory diagnostic and screening tools are currently available.

• Symptoms may overlap with other neurological and psychiatric problems such as organic syndromes (e.g. drug-induced psychoses, delirium and dementia), psychotic mood disorders and personality disorders.

It would clearly be desirable to identify a diagnostic tool for schizophrenia that is highly specific, and highly sensitive. Of importance the marker should signify the disease early in its course, as there is evidence that delays in diagnosis and intervention lead to a poorer prognosis. In addition, a method that is cost-effective and non-invasive would be of added value [2,4].

## Objective of the review

The use of biological markers in medicine has come a long way with advances in the fields of pathology, biochemistry and most notably genetics. For example, prevalent and debilitating diseases such as heart failure can be diagnosed with high sensitivity and specificity by measurement of levels of B-natriuretic peptide (BNP). Certain types of cancer can be screened for and monitored by specific tumor markers. Genetic diagnoses can be obtained for cancers, and numerous other diseases from Parkinson's to hypertrophic cardiomyopathy.

In psychiatry, biomarker research is more problematic due to the difficulty in illuminating the pathophysiology of psychiatric disorders. Despite this, extensive research on the genetics and proteomics of neurodegenerative disorders such as Alzheimer's disease (AD) is making headway [5]. This article is an update of the paper by Lakhan [2] and reviews the current status of biomarker research for schizophrenia, with emphasis on proteomics and genomics.

## Biomarkers

**Figure 1:**
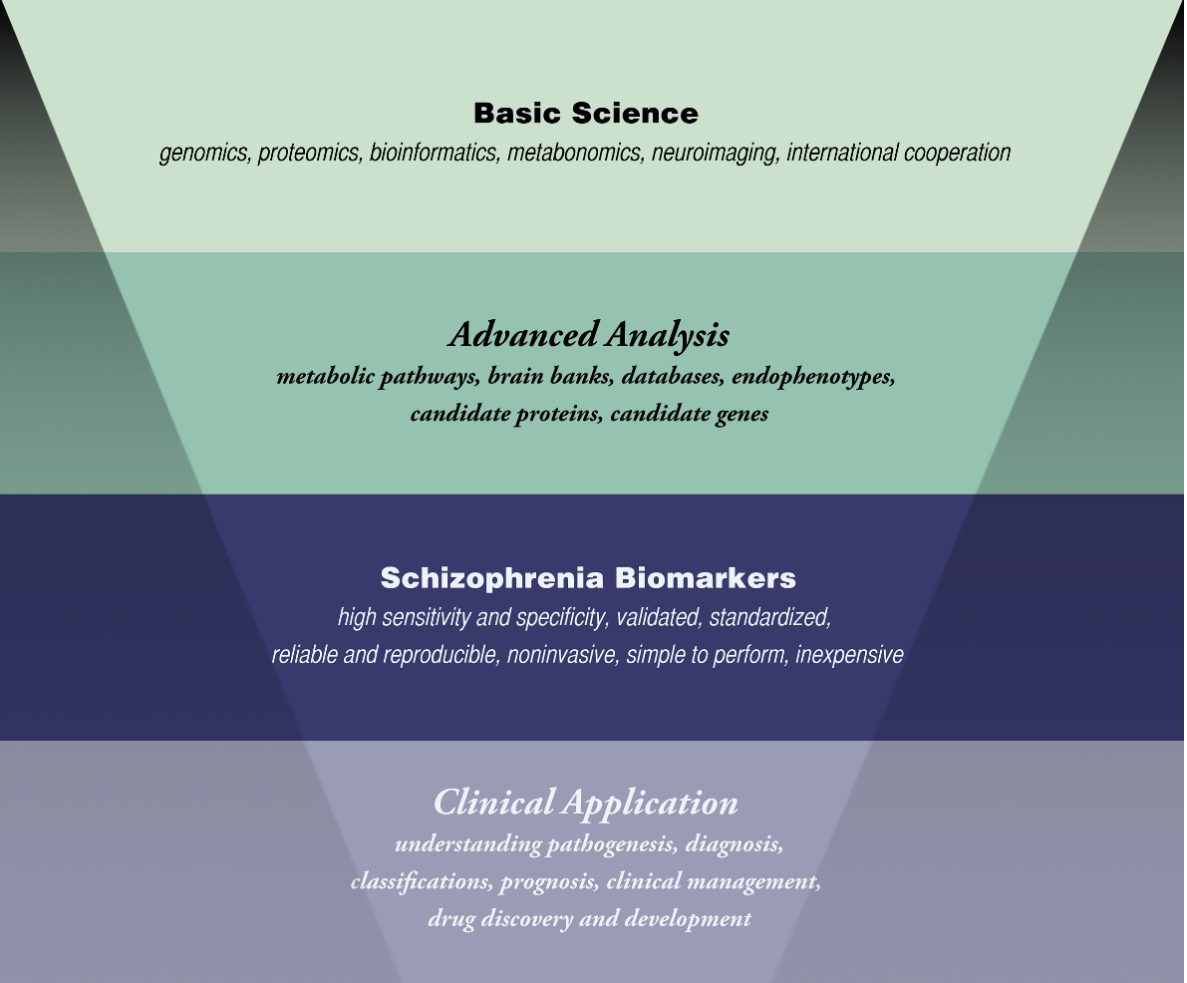
**The pathway to schizophrenia biomarker discovery and clinical applications**.

Biomarkers may be in the form of genes, proteins and other molecules, or morphological characteristics. Depending on the information they can provide, biomarkers may be used in diagnostics as prediction tools (e.g. subclinical markers, risk or vulnerability markers), or as diseases signatures (e.g. disease markers, stage or progression markers) (see Figure 1).

Although the pathophysiology of schizophrenia remains unclear, there is an increasing body of evidence that several molecular pathways are involved. Most findings point to the direction of malfunctioning of the glutamate pathway [6]. It is hoped that these molecular processes are mirrored in easily detectable biomarkers. However, this is not the case thus far.

### Genes as biomarkers: from genes to symptoms

Genetic studies have attempted to identify genes that predispose an individual to certain diseases. Examples of currently used genetic biomarkers are the breast cancer susceptibility gene 1 (BRCA1) or breast cancer susceptibility gene 2 (BRCA2).

The genetic basis of psychiatric disorders has, thus, far proven to be more complex than many other diseases. Though there is evidence, mostly from simple familial studies that there are degrees of inheritance in many psychiatric problems, the relationship between genes and phenotype, and furthermore the penetrance and expressivity of candidate genes is difficult to determine. For example, individuals showing schizotypal behavior traits but not overt psychotic symptoms may have a similar genetic profile to a diagnosed schizophrenic patient, but the way the genes manifest themselves can be altered by developmental and environmental factors, i.e. expressivity. That is assuming that schizophrenia lies on the end of a continuum with schizotypal personality disorder.

According to Riley et al. [6], schizophrenia is not genetically determined but rather genetically mediated. Multiple genes of varying effects interact with each other and with environmental factors to cause the wide spectrum of schizophrenic symptoms.

#### Association studies: SzGene database

As previously reviewed by Lakhan [2], genes linked to schizophrenia are found in several chromosomal regions. A big step in schizophrenia genomics is the creation of the SchizophreniaGene (SzGene) database by Allen and colleagues [7] to collate all association studies on schizophrenia. It is estimated that over 1000 schizophrenia genetic association studies have been conducted to date and SzGene aims to facilitate interpretation of findings across geographical areas. It also facilitates meta-analyses of largely inconsistent but still valuable results in schizophrenia genomics, including allele-based meta-analyses for polymorphic genotype data. These meta-analyses have already identified more than 20 different candidate genes, both well-known as well as novel genes [8].

Using the SzGene database, Sun et al. [9] conducted a survey of over 2000 linkage studies involving 539 candidate genes for schizophrenia. Using a ranking system based on combined an odds ratio method, the authors came up with a set of top ranking genes that future studies may be able to use as a working blueprint. Among those most highly ranked are DISC1, DTNBP1, COMT, DAO, RGS4, NRG1, and GRM3. Below we will look closely at two of the most promising gene markers for schizophrenia.

#### The DISC1 gene

One of the most promising of the many risk gene marker candidates is the disrupted in schizophrenia 1 (DISC1) gene [10,11]. The DISC1 linkage was initially observed in extended Scottish families but has now been replicated in Finnish, American, Japanese, and Taiwanese several populations [12].

A SzGene database search listed 24 schizophrenia-association studies for DISC1 from 2001 to February 2008. Of these, 15 were ethnic-based studies and 9 were family-based. However, most of the studies were based on Caucasians and Asians; other ethnic groups are underrepresented. Fifteen studies reported positive association of DISC1 with schizophrenia.

Evidence, mainly from animal models, supporting the potential of DISC1 gene as a biomarker, is mounting. Associations have been found between mutant DISC1 genes and neurocognitive deficit symptoms specific for schizophrenia and bipolar disorders [12]. DISC1 is present in regions (i.e. cerebral cortex and hippocampus) of the brain that shows abnormality in schizophrenia patients; aberrant DISC1 expression results in reduced volume of frontal cortical gray matter [12].

The function of the DISC1 gene or its mechanistic role in schizophrenia is still not fully known and current research is focusing on filling this knowledge gap.

#### DTNBP1

Another potentially valuable gene marker for schizophrenia is the dystrobrevin-binding protein 1 (DTNBP1), also known as dysbindin. A SzGene database search for DTNBP1 revealed 28 case-control association studies and 16 family-based studies between 2002 and April 2008. Most studies involved Caucasians, followed by Asians, though a few studies on mixed ethnicities were also registered. Of a total of 44 studies, only 18 reported positive association with schizophrenia.

DTNBP1 is considered to be a modifier gene and has shown strong evidence of illness modification involving negative and cognitive symptoms [13]. It was found to interact with the interleukin 3 (IL3) gene and jointly contribute to schizophrenia risk [14].

Although the search for schizophrenia genetic markers seems to be making progress, the fact remains that there are big overlaps in the susceptibility genes between schizophrenia and other psychotic and mood disorders [15]. This problem of specificity needs to be resolved before these genes can be routinely used for genetic screening for schizophrenia.

#### Endophenotypes

It is believed the number of genes involved in a phenotype increases with the phenotype's complexity, thus, increasing the complexity of genetic analysis as well [16]. The fact that schizophrenia is a pleiotropic disorder multiplies the complexity several fold. The endophenotype approach in psychiatry aims toward a more tractable and objective diagnosis of psychiatric disorders using genetic analysis.

Endophenotypes are heritable trait-related deficits measurable by laboratory-based diagnostic tools rather than by clinical investigations [17]. An endophenotype may be neurophysiological, biochemical, endocrinological, neuroanatomical, cognitive, or neuropsychological symptoms and would include configured self-report data. Several candidate endophenotypes such as working memory, oculomotor function, sensory motor grating, and glial cell abnormalities have been linked to certain gene regions [16].

An example of using oculomotor function as a trait or state marker in neurological disorders has been described by Chen et al. [18]. Dysfunction in visual processes such as eye tracking, visual backward masking, motion perception, and contrast detection were observed in patients with schizophrenia and bipolar disorder.

Bender et al. [19] reviewed common early recognition instruments for psychosis in the context of endophenotypes that included P300, P50 sensory gating, mismatched negativity, smooth pursuit eye movements.

Endophenotypes in the form of cognitive neuroimpairments such as prospective memory deficit [20,21], and visual and verbal memory deficits [22] have also been described for patients with schizophrenia.

In summary, in the field of genomics, progress is being made in several fronts. In the case of association studies, all ethnic groups should be represented for data to be more meaningful. Currently, the number of schizophrenia candidate genes is too large; priority should be given to identifying the most relevant genes, especially those linked to endophenotypes. It has also been suggested that whole-genome association studies can reveal the genetic modifiers of schizophrenia [13].

### Proteomics: from genes to proteins

It is not the presence of certain proteins per se that make them markers but rather their expression, the shifts in expression as well as their state. In many cases, however, it is not just one protein but a set of proteins that is indicative of a disease. Using breast cancer as example, the most common diagnostic molecular biomarkers for this disease are estrogen receptor (ER), progesterone receptor (PR), and HER2/neu proteins.

A primary challenge in the search for biomarkers is easy access to sufficient amounts of high-quality tissue or body fluids [23]. Sources of biomarkers are the blood, urine, cerebrospinal fluid (CSF), and certain tissues.

#### Biomarkers in blood

Diagnostic tests using blood sample is standard in clinical practice. As source of biomarkers, it has the advantage of easy and standardized collection procedure, and can be available in sufficiently large volumes. However, blood-based specific biomarkers for schizophrenia are still elusive.

Bibl et al. [24] report that the ratio of Aβ1–38/Aβ1–40 peptides in the plasma of patients with dementia may be a potential diagnostic tool. Another study [25] identified 18 signaling proteins in blood plasma of patients with AD. These proteins were used in blinded identification with almost 90% accuracy.

Serum levels of the inflammatory markers sTNFR1 and sTNFR2, (soluble receptors of the tumor necrosis factor) were found to be higher in chronic institutionalized patients with schizophrenia than in control individuals. However, no correlation with symptom severity was found [26].

Dietrich-Muszalska and Olas [27] looked at the role of oxidative stress in schizophrenia patients as manifested in blood platelet aggregation in response to known platelet agonists such as ADP and collagen. Collagen-stimulated platelet aggregation was significantly lower in the patient group than in the healthy controls. ADP-induced aggregation on the other hand, was significantly higher.

In another study [28], the activity of the platelet antioxidative enzyme superoxide dismutase (SOD), and the levels of thiobarbituric acid reactive species (TBARS) were measured as oxidative stress indicators. Results suggested an enhanced generation of reactive oxygen species and significantly lower SID activity in schizophrenia patients compared to healthy controls.

The telomere length in the peripheral blood lymphocytes (PBL) of individuals with schizophrenia has been observed to be significantly reduced. In a follow-up study, Porton et al. [29] quantified the activity of the enzyme telomerase in PBL and found a nominally significant decrease in telomerase activity in individuals with schizophrenia compared to unaffected individuals.

#### Biomarkers in urine

Urine has proven to be an invaluable source of biomarkers for many urological, gynecological and metabolic disorders and infections. It has the advantages of being available in large amounts, collection is non-invasive, and it remains stable for extended periods [29]. On the downside, protein content in urine is highly variable and relatively low, and salt content is high compared to other body fluids [30,31]. Except for very few studies, this source has proven to have limited success in the neuroproteomics of psychiatric disorders.

Indicators of oxidative stress are detectable in the urine. Significantly increased levels of isoprostanes were observed among schizophrenia patients relative to the controls, as measured by isoprostane-8-epi-prostaglandin F(2alpha) (8-isoPGF(2alpha)) concentrations in the urine [32]. Oxidative damage by free radicals and the measurement of isoprostanes in the urine of AD patients have been previously described [33].

Another study [34] measured concentrations of biopyrrins, metabolites of bilirubin oxidation, in the urine of patients with schizophrenia and depression. Biopyrrin values of schizophrenia patients significantly correlated with Brief Psychiatric Rating Scale (BPRS) scores. For patients with depression, biopyrrin values correlated significantly to the Hamilton Depression Rating Scale (HAM-D) scores.

#### Biomarkers in the cerebrospinal fluid

Because of its proximity to the affected tissues of the nervous system, it seems logical that the chemical properties of the cerebrospinal fluid (CSF) would more closely reflect pathophysiological alterations caused by neurological disorders than any other body fluids.

The use of CSF as standard biomarker source is however, a subject of controversy. There are those who perceive the sampling procedure through lumbar puncture as invasive and risky for the patient. CSF collection is not a standard clinical procedure in many countries and may therefore require obtaining informed consent, a process that is complicated by the mental status of the patients concerned [35,36]. Others claim that CSF can be very variable depending on collection procedure, storage, and time of the day [23]. However, there are also studies which report lumbar puncture to be safe and acceptable even in patients of mixed cognitive status [37,38].

Still, CSF-based neurochemical diagnostics look very promising in many neurological disorders including AD [5,39] and amyotrophic lateral sclerosis (ALS) [40]. Increased tau and decreased Aβ42 have been validated as AD disease markers [41]. In a recent study, Zhang et al. [42] reported a CSF-based multianalyte profile that can distinguish between AD and Parkinson disease.

Huang et al. [35] detected characteristic profiles of peptides and proteins in CSF of schizophrenia patients which are potentially specific for schizophrenia. Using the latest mass spectrometric approaches (see methodological review [2]), they detected significant changes between protein/peptide profiles of drug-naïve schizophrenia patients compared to demographically matched healthy controls. The key fragments were a 40-amino acid long VGF-derived peptide sequence, a transthyretin protein cluster, and another partly characterised smaller cluster related to transthyretin. A simple and controlled method for CSF-biomarker discovery is described by Huang et al. [36].

To test for specificity, the alterations were compared with CSF samples from patients with similar neurological conditions. They were found to be specific for patients with schizophrenia, slightly similar for patients with depression but very distinct from patients with OCD or AD. The authors [36] report 95% specificity and 80 to 88% sensitivity in the two sets of experiments. Limitations of the study include the small number of samples especially those of non-schizophrenia psychosis patients.

#### Biomarkers in brain tissues

Miller et al. [43] looked at the metabolites of the kynurenine pathway in post-mortem brain tissues of patients with schizophrenia and bipolar disorder. The precursor tryptophan was higher in the schizophrenia group; the end-product nicotinamide did not differ significantly between patient groups and controls. In a follow-up study, Miller and Dulay [44] looked at niacin receptors and reported that the protein for HM74A receptors was significantly lower in the schizophrenia group, while transcription for the protein increased. No significant differences were observed between the bipolar disorder group and controls. The studies presented some potentially highly specific biomarkers than need to be confirmed in biofluids.

One of the criteria for an ideal diagnostic biomarker for AD is validation against autopsy-proven cases [4]. However, Ranganathan et al. [45] report big differences between the protein profile of CSF from live patients with amyotrophic lateral sclerosis (ALS) and that of post-mortem brain tissues.

A significant step forward in biomarker research is the establishment of brain banks or biorepositories that collect post-mortem brains and CNS tissues for research purposes [46-50] although the initiative is rife with controversies regarding legal and bioethical issues.

In summary, CSF is probably the most promising source of biomarkers, followed by blood. There is, however, a great need for standardization of study designs and methods. Comparison of proteins found in the blood and the CSF would be a step in the right direction. There are already indications that certain biomarkers are transported from plasma to CSF and vice versa [51] but these need to be confirmed by more extensive studies.

### Bioinformatics: bridging genomics and proteomics

Current studies are focusing on protein expression and protein-protein interactions (PPI) around certain genes to bridge the knowledge gap between genomics and proteomics. Through bioinformatics, a network of protein-protein interactions around the DISC1 gene was generated, the so-called "DISC1-Interactome" [11,52]. The interactome came up with 2 potential DISC1-related protein candidates, the NDEL1 (nudE nuclear distribution gene E homologue-like 1) and phosphodiesterase 4B (PDE4B), a cAMP signalling-specific phosphodiesterase [52]. NDEL1 may function both as a cysteine protease and a key centrosomal structural protein. With its interaction with DISC1, it is said to significantly influence the risk for schizophrenia [53]. PDE4B is an independently identified risk factor for schizophrenia [54].

Like DISC1, a similar interactome for DTNBP1 has now also been generated [55].

Researchers in Taiwan [56] have recently set up PhosphoPOINT, an open-access comprehensive human kinase interactome and phospho-protein database. It basically integrates the characteristics of the abovementioned proteins, their PPI datasets, substrates, interactions, gene expression and ontology, as well as their single nucleotide polymorphisms (SNPs), and their role in human diseases. Linked phenotypes in the database include schizophrenia and hypertension.

### Metabonomics: metabolic pathways and metabolites

The controlled metabonomics study by Holmes et al. [57] investigated the metabolic profiles of CSF collected from patients with first-onset schizophrenia not treated with any antipsychotic drugs. The metabolic state of CSF was determined through glucoregulatory processes and their metabolites. Glucose levels in the CSF of drug-naïve patients show highly significant increased glucose levels compared to health control subjects. These elevated CSF glucose levels were not reflected in blood glucose levels, indicating a central nervous system-specific process.

Subsequent tests by the same group of researchers showed that short-term therapy using atypical antipsychotic drugs has the tendency to normalize of the CSF metabolic profile among patients with schizophrenia. The normalization was detectable well before clinical symptoms overtly improved. This study suggests that the products of glucometabolic process in the CSF can be used as a disease marker for first-onset paranoid schizophrenia.

Limitations of the study are the small number of patients in some of the treatment groups and the popular use of cannabinoids among patients with schizophrenia, which may have affected metabonomic measurements.

Metabonomics is a relatively new field of research. It is perceivable that data on metabolic processes can contribute to the understanding of the pathophysiology of schizophrenia.

### Neuroimaging: from genes to morphology and function

Neuroimaging has been invaluable in studying the structure and function of the brain. It also has a great potential for the diagnosis and treatment and neuropsychiatric disorders.

Using cognitive paradigms, functional magnetic resonance imaging (fMRI) studies have shown changes in cerebellar activity in patients with schizophrenia, anxiety disorders and dementia (see review [58]).

In a twin pair study [59], abnormalities in proton magnetic resonance spectroscopy (1H MRS) neurometabolites glutamate, creatine plus phosphocreatine (Cr), glycerophosphocholine plus phosphocholine (Cho), and N-acetylaspartate were not present in control individuals but present in both schizophrenia-affected and unaffected twins, although the levels were significantly higher in the affected twin. This indicates that these abnormalities can be used as trait markers as well state markers of the disorder.

Kubicki et al. [60] observed significant reduction in interhemispheric brain connectivity in the corpus callosum of individuals with schizophrenia using the diffusion tensor imaging techniques.

Recent research studies used neuroimaging techniques in linking genes to brain morphology and function. One such study by Windemuth et al. [61] identified endophenotypes associated with schizophrenia. They found 3 single nucleotide polymorphisms in certain genes were significantly associated with fMRI activity.

Giedd et al. [62] proposed that changes in brain morphology over time as measured by neuroimaging techniques may be used as endophenotypes for neurological disorders. These so-called brain trajectories may be used as markers that bridge the gap between genes and behavior.

Using MRI, Takahashi et al. [63] investigated the association between brain morphology and gene polymorphism. Their results indicate that high-risk genotypic combinations affect brain morphology, particularly the posterior hippocampus.

These latest developments show that neuroimaging is invaluable in linking brain function and activity to form. However, more studies focused on the schizophrenic brain and how it is linked to disease-specific genes or gene regions are needed to link trait markers to biomarkers.

## Discussion and conclusion

This article attempts to review the most significant findings in the field of schizophrenia biomarker research during the last two years. While none of the developments described here qualify as biomarker discovery, many will likely serve as stepping stones towards that goal. To summarise the major achievements:

• There has been a rapid increase in schizophrenia research; current publication rate as estimated by SzGene databases is 90 papers a week [7].

• Genetic studies have definitely made headway in screening for candidate schizophrenia genes in association studies. Endophenotypes or intermediate phenotypes are useful in the understanding the pathway from genes to behavior.

• Schizophrenia proteomics lags behind compared to other neurological disorders such as AD. However, progress has been made in identifying candidate protein and metabonomic markers. CSF as a source of disease markers looks especially promising as demonstrated in the case of AD.

• Neuroimaging is also making progress in linking genotypes to brain morphology, supporting the endophenotype approach.

• International cooperation in the form of networks, open access databases and brain banks is facilitating comparison and verification of results.

However, there is still a long way to go before biomarkers become part of the standard clinical care for schizophrenia patients. Before we get there, there are several things that need to be improved upon, as follows:

• We need to conduct larger studies for more statistical power.

• Candidate biomarkers need to be tested for sensitivity and specificity to resolve overlaps with related disorders.

• More association studies are needed, especially those looking at other ethnic groups besides Caucasian.

• Methods for sample collection, storage, and sample processing of biofluid sources of biomarkers, especially CSF, need to be standardized.

## Abbreviations

1H MRS: proton magnetic resonance spectroscopy; 8-isoPGF(2alpha): isoprostane-8-epi-prostaglandin F(2alpha); ALS: amyotrophic lateral sclerosis; BNP: B-natriuretic peptide; BPRS: Brief Psychiatric Rating Scale; CDC: Centers for Disease Control and Prevention; Cho: glycerophosphocholine/phosphocholine; Cr: creatine/phosphocreatine; CSF: cerebrospinal fluid; SzGene: SchizophreniaGene; DISC1: Disrupted-In-Schizophrenia 1; DTNBP1: dystrobrevin-binding protein 1; ER: estrogen receptor; fMRI: functional magnetic resonance imaging; HAM-D: Hamilton Depression Rating Scale; IL3: interleukin 3; MRI: magnetic resonance imaging; NDEL1: nudE nuclear distribution gene E homologue-like 1; OCD: obsessive-compulsive disorder; PBLL: peripheral blood lymphocytes; PDE4B: phosphodiesterase 4B; PPI: proton pump inhibitor; PR: progesterone receptor; SNP: single nucleotide polymorphism; SOD: superoxide dismutase; TBARS: thiobarbituric acid reactive species.

## Competing interests

The authors declare that they have no competing interests.

## Authors' contributions

SEL and AK participated in the preparation of the manuscript. All authors read and approved the final manuscript.
